# Editorial: Micro/nano motors and robots

**DOI:** 10.3389/fbioe.2022.1074467

**Published:** 2022-11-11

**Authors:** Renfeng Dong, Yuepeng Cai, Tailin Xu, Beatriz Jurado Sánchez

**Affiliations:** ^1^ School of Chemistry, South China Normal University, Guangzhou, China; ^2^ School of Biomedical Engineering, Health Science Center, Shenzhen University, Shenzhen, China; ^3^ Department of Analytical Chemistry, Physical Chemistry, and Chemical Engineering, Universidad de Alcala, Alcala de Henares, Madrid, Spain

**Keywords:** nanotechnology, micro/nanomomotor, motion control, micro/nanorobots, application

Micro/nano motors and robots are micro/nano-sized devices that have controllable motion and can perform specific tasks in a micro-environment. Due to their attractive properties, they show considerable potential in various fields ranging from biomedical applications to environmental remediation. This Research Topic covers a collection of articles on cutting-edge developments in motion control and biomedical environment applications of micro/nano motors and robots.

Efficient motion control is critical for the practical applicability of micro/nano motors and robots. Unlike passive micro/nanoparticles, the most attractive advantage of micro/nano motors or robots are their controllable motion performance Motion control is significant for groups of motors or robots since it means higher work efficiency and stronger transport power. Inspired by the biological collective behaviors of nature, Zhang et al. present a strategy that reconfigures paramagnetic nanoparticles into a vector-controlled microswarm with 3D collective motions by programming sawtooth magnetic fields. It can be flexibly switched from a horizontal swarm to a vertical stand and hover *in situ*, and the wheel-like swarms are endowed with multi-modal locomotion and load-carrying capabilities Li et al.

For a single motor or robot, speed and directional control are the two most important properties. Indeed, with strong propulsion, the self-stirring effect of the microenvironment generated by the motors or robots will greatly improve their contact efficiency with a given target, thereby improving the efficiency, especially in biomedical applications, such as cell detection, drug delivery, DNA sensing, *etc.* Peng and coworkers creatively demonstrated a new advantage of the strong propulsion that the physical movement and the force of actively moving micro/nano motors can open up new possibilities for solving biomedical issues, such as T cell regulation. They developed Pd/Au nanomotors with a spiky morphology which exhibited continuous locomotion in the cellular biological environment, the active motors’ generate physical cues (force and pressure) are sensed by mechanosensitive ion channels of T cells and trigger Ca^2+^ influx and subsequent activation Fu et al.

In addition to the utilizing propulsion, compared to traditional drug delivery systems that usually rely on passive diffusion, efficient direction control of micro/nano motors or robots means active targeted delivery which also plays a key role in biomedical applications. In this context, Hua et al. have illustrated magnetic-driven hydrogel microrobots as a drug delivery system for drug delivery directly to the tumor site by magnetic field regulation to enhance the drug efficiency and reduce the side effects. Meanwhile, the selective inhibition of this system could be easily controlled by programming the strength of the magnetic field Mu et al.

For drug delivery, many other excellent micro/nano motors or robots have been successfully developed. Therefore, the most recent developments of micro/nanorobots in “motile-targeting” drug delivery have been well summarized by Zhang et al. In this review, after a brief introduction to traditional tumor-targeted drug delivery strategies and various micro/nanorobots, the representative applications of micro/nanorobots in “motile-targeting” drug delivery are systematically streamlined in terms of the propelling mechanisms.

In addition to biomedical applications, micro/nano motors and robots also show great potential in environmental applications, such as degradation, absorption, sensing, etc. However, long-distance work in the environmental field is still a challenge for micro/nano motors and robots due to their nano/micro scale and limited propulsion. In this Research Topic, Wang et al. proposed a creative robot system that can realize long-distance transportation and drop the Janus microrobots to a targeted destination by a flapping-wing micro aircraft (FWMA), and then, the released Janus microrobot can be efficiently propelled and navigated by magnetic field regulation for oil absorption and organic pollutants process.

